# Supplementary figures and images for: Establishing standardized conditions for clinically available sound-localization tests: A multicenter approach
Source: PLoS One. 2025 Aug 14;20(8):e0327124. doi: 10.1371/journal.pone.0327124 (PMC12352838; doi:10.1371/journal.pone.0327124)

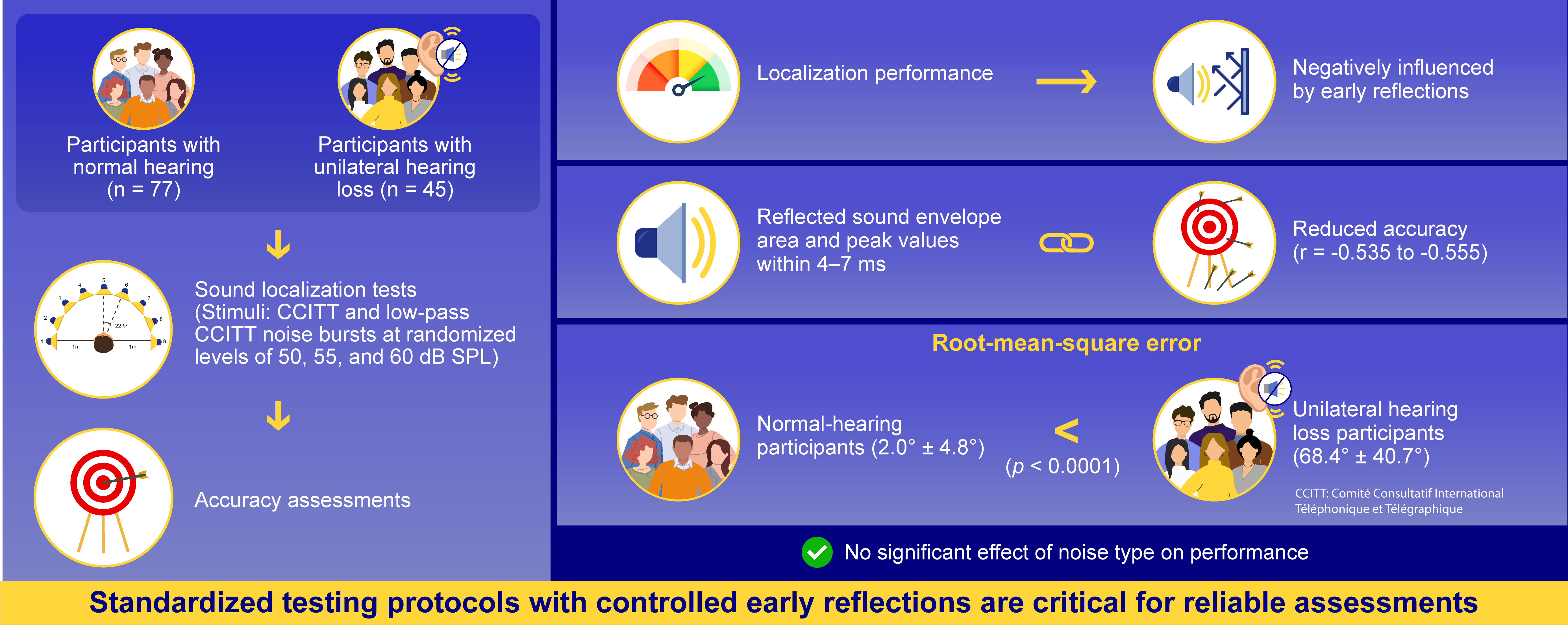

Supplement: S1 Fig — (TIF) [file pone.0327124.s003.tif]
