# Supplementary material for: Establishing standardized conditions for clinically available sound-localization tests: A multicenter approach
Source: PLoS One. 2025 Aug 14;20(8):e0327124. doi: 10.1371/journal.pone.0327124 (PMC12352838; doi:10.1371/journal.pone.0327124)
Supplement: Attachment — Submitted filename: Response_to_Reviewers.docx [file pone.0327124.s009.docx]

Review Comments to the Author

Please use the space provided to explain your answers to the questions above. You may also include additional comments for the author, including concerns about dual publication, research ethics, or publication ethics. (Please upload your review as an attachment if it exceeds 20,000 characters)

Reviewer #1: The study explored localization abilities of sounds in the azimuthal dimension in order to establish standardized acoustic conditions for sound localization assessments in unilateral hearing loss and control individuals.

The multicenter approach is a plus-value. The method is clear. Establishing standardized tests is a key objective. I think the Results section could be improved, as well as the discussion. I think there is a lack of references in the introduction (eg. my comment 5).

I noticed some mistakes (eg. sentences written twice). The details are reported below in my comments.

It seems that the data availability statement does not fit with the requirements of Plos ONE. They state “No – some restrictions will apply” without explaining the exceptional situation as asked in the guidelines. I do not think this is a reason to reject the paper but I think it is necessary to explain why data is available only upon request to the author Tadao Yoshida.

General comments:

1) It is unclear whether the study was financially supported. In the Financial Disclosure you mention that “the author(s) received no specific funding for this work” but in the Acknowledgment section you mention “This research was supported by AMED under Grant Number JP24dk0310128.”. Could you clarify this please?

Thank you for pointing out this discrepancy regarding funding. We apologize for the lack of clarity.

To clarify, this study was indeed supported by the Japan Agency for Medical Research and Development (AMED) under Grant Number JP24dk0310128. The original statement in the Financial Disclosure section, “the author(s) received no specific funding for this work,” was incorrect and has been deleted.

We have updated the Financial Disclosure section to reflect this as follows:

Revised Financial Disclosure:

"This study was supported by the Japan Agency for Medical Research and Development (AMED) under Grant Number JP24dk0310128."

2) In the figure, I think it would be clearer if you also mention the azimuth position of the loudspeakers 1CH/2CH […]/ 9CH.

We have added the azimuth positions of the loudspeakers (ch 1/ch 2 […]/ch 9) to Figures 1, 4, and 5 to enhance clarity and spatial understanding for the readers. Please note that the figure numbers have been updated based on the suggestions from Reviewer 2, and the figures referenced in the revised manuscript may differ from the original numbering.

3) Please clarify the data availability statement to fit with the requirements of the journal.

Thank you for pointing this out. We have revised the Data Availability Statement to align with the journal’s requirements. Additionally, we have uploaded the relevant dataset as a supplemental file for transparency and accessibility.

Specific comments :

1) Lines 101-105 : You mention ILD and ITD as acoustic cues for spatial hearing. Please mention Interaural Phase Difference (IPD) which is an important cue when ITD and ILD are not sufficiently reliable.

The interaural phase difference (IPD) is an important auditory spatial cue that becomes particularly useful when interaural time differences (ITD) and interaural level differences (ILD) are not sufficiently reliable. The IPD refers to the difference in the phase of a sound wave arriving at each ear, and it is especially effective at lower frequencies where phase information is more discernible and ITD and ILD cues can be ambiguous or diminished [2,3] (lines 81–85).

[2] Blauert, J. Spatial hearing: The psychophysics of human sound localization. Revised ed. Cambridge, MA: MIT; 1996.

[3] Zirn S, Arndt S, Aschendorff A, Laszig R, Wesarg T. Perception of interaural phase differences with envelope and fine structure coding strategies in bilateral cochlear implant users. Trends Hear. 2016;20: 233121651666560.

2) Line 110 : If I have well understood what you mean by “frequency intensity”, you refer to the energy in a frequency band. I think it would be clearer if you use the term “energy” or “spectral component” instead of “frequency intensity”.

Thank you for your helpful comment. You are correct in your interpretation — by “frequency intensity,” we intended to refer to the energy within a given frequency band. To improve clarity and accuracy, we have replaced the term “frequency intensity” with “spectral component” throughout the manuscript (lines 89, 90).

3) Lines 114- 115: you write “Thus, since these three elements (ITD, ILD, spectral cues), and changes in sound pressure due to the head-shadow effects are important in directional perception […]”. Can you explain why you mention separately ILD and the changes in sound pressure du to the head-shadow effects?

We agree that mentioning both ILD and the changes in sound pressure due to the head-shadow effect may have been redundant, as ILD largely reflects the acoustic attenuation caused by the head shadow. To clarify this point and to improve the scientific accuracy, we have revised the sentence as follows:

“Thus, since these four elements (ITD, ILD, IPD, and spectral cues) are important in directional perception, a test method that reflects these detection capabilities is needed for accurate sound-localization testing” (lines 94-96).

This revision eliminates the redundant mention of head-shadow effects and incorporates IPD, which plays an important role when ITD and ILD are less reliable, particularly at low frequencies or in complex acoustic environments.

4) Lines 118-120 : You state “In general, sound-localization test results are worse in patients with unilateral hearing loss (UHL) than in healthy participants, and cognition on the side of the hearing-impaired ear is worse than that on the side of the normal-hearing ear. “ . I think there is a lack of references. Please mention references for sound-localization performance lower in patients with UHL than non-impaired. Also I think you could specify what you are referring to when you say “cognition” (for instance, give examples of tasks and references).

As suggested, we have added appropriate references to support the statement that sound-localization performance is poorer in individuals with unilateral hearing loss (UHL) compared to that in normal-hearing individuals (lines 99–102). The following references have been cited in the revised manuscript:

[6] Parisa A, Reza NA, Jalal SS, Mohammad K, Homa ZK. Horizontal localization in simulated unilateral hearing loss. J Audiol Otol. 2017;22: 39–44.

[7] Nelson E, Reeder RM, Holden LK, Firszt JB. Front- and rear-facing horizontal sound localization results in adults with unilateral hearing loss and normal hearing. Hear Res. 2019;372: 3–9.

In addition, we have revised the word “cognition” to “recognition” in this sentence for greater clarity. The revised sentence now reads:

“In general, sound-localization test results are worse in patients with unilateral hearing loss (UHL) than in healthy participants, and recognition on the side of the hearing-impaired ear is worse than that on the side of the normal-hearing ear” (line 98).

5) Lines 131 and 136: The ages of UHL patients and control individuals are different. Did you test the effect of age on performance? Auditory perception capabilities (comprising auditory localization) tend to decrease with age. I think you could mention it in the discussion.

We thank the reviewer for highlighting this point. To evaluate the potential influence of age, we conducted an analysis of covariance (ANCOVA) with age as a covariate. The group difference between UHL and control individuals remained significant (p < .001) for every loudspeaker, indicating that age did not explain the observed differences in performance. We have added this clarification to the Results and Discussion sections (lines 316–320; 335–340).

6) Lines 275-276: It seems something is wrong with the sentence “A positive correlation of the envelope area with the differences between the number of correct responses between the left and right sides”. Is there a verb missing ? Please check this

Thank you for pointing this out. You are correct that the original sentence was unclear due to the absence of a main verb. We have revised the sentence for grammatical clarity and readability. The corrected sentence now reads:

“There was a positive correlation between the envelope area and the difference in the number of correct responses between the left and right sides” (lines 266–267).

7) Lines 318-379: the following sentence is already written line 299 : “In cases of UHL, the side of the worse-hearing ear was considered to be ch 9, and if the left ear was the worse-hearing ear side, the results were analyzed symmetrically.” Please check this

Thank you for your careful reading. While the sentence at line 299 (“In cases of UHL, the side of the worse-hearing ear was considered to be ch 9, and if the left ear was the worse-hearing ear side, the results were analyzed symmetrically.”) appears similar to the sentence in lines 318–379, we would like to clarify that the line 299 sentence appears within the figure legend. As such, it serves to explain the figure independently and does not constitute redundancy within the main text. We believe it is important to retain this information in both locations to ensure clarity for readers who refer to the figure and those who follow the main text.

8) In the Result section, I think it is not enough clear whether the first results concern control individuals and UHL patients or just control individuals. Could you clarify this please? Also, I think it is not enough clear whether you tested the same predictors for the control group and the UHL patients. You mention effects for the control group but it is not clear if you this effects are also present in the UHL group. Is there interactions with the group (patients versus controls)?

We thank the reviewer for this insightful comment. To address the issue, we revised the Results section to clearly distinguish the analyses conducted in each group by adding the following subheadings: Control study (measurement of reflected sound), Control study (sound-localization test), and UHL study (sound-localization test).

Furthermore, we have added the following sentence to the Materials and Methods section under the Sound-localization test heading to clarify the consistency between groups:

“Both groups (control and UHL) underwent sound-localization testing under the same acoustic conditions and procedures. The results were analyzed using the same statistical model and predictor variables to ensure consistency and comparability between groups” (218–221).

We also examined whether there were any interaction effects between the group (control vs. UHL) and the predictors. Both groups showed significant differences in RMS at all loudspeaker positions. Therefore, interaction effects were considered negligible.

9) Line 332: Do you have explanations for the non-effect of the noise type on performance ? I think it could be clearer if you mention again the low-pass filter (1500 Hz).

Thank you for your insightful question. We agree that further clarification is needed regarding the non-significant effect of noise type on performance.

In our study, the low-pass CCITT noise was filtered at 1500 Hz, which primarily removes higher-frequency spectral cues. However, sound-localization performance both in participants with normal hearing and in those with UHL is thought to rely more heavily on low-frequency ITDs and IPDs in such conditions. Therefore, the removal of high-frequency components may not have significantly disrupted the critical localization cues utilized by participants in either group.

We have added this explanation and clarified the 1500 Hz low-pass filtering in the revised manuscript (lines 340–345).

10) Line 339. In the discussion you mention “channels 1, 2, 8 and 9”. I think the discussion would be improved if you mention also the azimuth position in degree. Therefore the reader can easily linked the positions of your loudspeakers with the positions mentioned in the discussion (line 366 for instance).

Thank you for your helpful suggestion. We agree that including the azimuth positions would enhance clarity. We have revised the sentence to read:

“This association was most prominent in channels 1 (-90°), 2 (-67.5°), 8 (67.5°), and 9 (90°), where early reflections exhibited a strong negative correlation with the localization accuracy” (lines 350–352).

11) Line 359-360: In the sentence “Some facilities demonstrated more than 10 discrepancies in the number of correct responses for loudspeakers in symmetrical positions”, please mention the total number of responses (eg 10 on how many?).

Thank you for your comment. To clarify, the "more than 10 discrepancies" refers to the number of mismatched correct responses out of 21 total responses per channel. We have revised the sentence to improve clarity as follows:

“Some facilities demonstrated more than 10 discrepancies (out of 21 responses) in the number of correct responses for loudspeakers in symmetrical positions, primarily distributed between channels 1(-90°) and 9 (90°), and channels 2 (-67.5°) and 8 (67.5°)” (lines 371–374).

12) Lines 411-413: I think the readers could be interested in practical examples mentioned here on how to reduce the initial reflections (eg, absorbing materials..).

Thank you for your valuable suggestion. We agree that providing practical examples for reducing early reflections would enhance the applicability of our findings. Accordingly, we have revised the sentence as follows:

“These findings suggest that reliable testing with minimal errors can be achieved by reducing the initial reflections, even in rooms of varying sizes or with different wall materials, for example, by installing sufficient sound-absorbing materials on immovable structures such as walls or pillars” (lines 424–427).

Reviewer #2: This paper describes a multi-center study to evaluate the effect of room reverberation on sound source localization for adults with vs. without unilateral hearing loss. Broadband and lowpass filtered noise was used. Results replicate two well-known findings: better localization accuracy at midline as compared to off midline, and deficits of localization for patients with unilateral hearing loss. Overall, similar results were obtained with the full-band and low-pass filtered noise. Of greatest interest here, performance differed between test sites. Acoustic analysis of sound recorded at each site indicate that performance is detrimentally affected by early reflections. Modifications of the test environments to reduce those reflections could presumably increase consistency of data cross sites.

The ultimate goal of this work, to configure and implement protocols for clinical assessment of localization, is valuable and motivated well. The research described here seems to be a reasonable first step towards this goal, with the caveat that some parameters are underspecified or non-standard. My primary comments have to do with the presentation of this work. There are some details missing that would be required to replicate this study, and the text does not provide enough information to understand the greater context of this work in the literature on spatial hearing.

I can guess why the stimulus parameters tested here were chosen – e.g., level rove to avoid reliance on a loudness cue and filtering to evaluate the relative roles of ITD and ILD – but I think readers would benefit from an explicit discussion of those parameters, including references to published work documenting those effects in other populations.

If I am following correctly, the full bandwidth and lowpass filtered stimuli were interleaved within a block of trials. If that is the case, then pre-trial uncertainty regarding spectral content could theoretically have interfered with the use of spectral shape as a cue to location. That seems like a point for discussion.

Thank you for your insightful comment. We would like to clarify that the full-bandwidth CCITT noise and the low-pass filtered CCITT noise stimuli were not interleaved within the same block of trials. Instead, they were presented in separate test sessions. Therefore, participants were not exposed to uncertainty regarding the spectral content of the stimuli within a given trial block; therefore, this factor is unlikely to have influenced their use of spectral shape cues for sound localization. We have added a sentence to the Materials and Methods section to make this point clear (lines 194–197).

The CCITT noise and low-pass CCITT noise conditions were tested in separate sessions. Participants were not exposed to randomized interleaving of different noise types within the same trial block, thereby avoiding uncertainty in spectral content during testing.

Detailed comments follow.

Line 108: These factors would introduce frequency-specific attenuation, but they wouldn’t increase or decrease the frequency of a sound. This paragraph was challenging for me to understand because the phrase “frequency intensity changes” was initially confusing. I would have found this easier to read if it had instead used the phrase “spectral shaping”.

Thank you for your helpful comment. You are correct in your interpretation — by “frequency intensity,” we intended to refer to the energy within a given frequency band. To improve clarity and accuracy, we have replaced the term “frequency intensity” with “spectral component” throughout the manuscript.

Line 119: It is not clear how cognition relates here. Should this be “recognition”?

We agree that the term “cognition” was not appropriate in this context and may have caused confusion. We have revised the sentence to use the term “recognition” instead, which more accurately reflects the intended meaning related to auditory processing performance on the side of the hearing-impaired ear. The revised sentence now reads:

“In general, sound-localization test results are worse in patients with unilateral hearing loss (UHL) than in healthy participants, and recognition on the side of the hearing-impaired ear is worse than that on the side of the normal-hearing ear.” (lines 97–99)

Line 121: Unilateral hearing loss does not always eliminate access to sound in the affected ear. Sometimes it just reduces access to sound or degrades the quality of that sound.

We agree that unilateral hearing loss does not necessarily eliminate all access to sound in the affected ear, but rather reduces it or degrades its quality, depending on the degree and configuration of the hearing loss. To reflect this more accurately, we have revised the sentence in question. The phrase “are unavailable for those with UHL” has been replaced with “are often substantially reduced or unreliable in individuals with UHL owing to limited or degraded input from the affected ear” (lines 100–102). We believe this revision better captures the nuanced auditory consequences of UHL.

Page 6, middle: It is not clear what units are used for defining pure-tone thresholds. If those values are in dB HL, then the normal-hearing controls don’t really have normal hearing in the traditional sense. Demographic information should also be provided for the NH group.

Thank you for pointing this out. We apologize for the lack of clarity regarding the units used for pure-tone thresholds. All pure-tone threshold values are expressed in dB HL. As stated in the Results section, the mean hearing level of the control participants who underwent pure-tone audiometry was 9.5 dB HL, which falls within the commonly accepted range for normal hearing. We fully agree that ≤ 25 dB HL is the conventional threshold for normal hearing. We have, therefore, revised the criterion throughout the manuscript.

Page 7, bottom: The position and selection of loudspeakers is not clear at this point in the text. Why was the speaker with the “smallest noise out of the three” the one that was selected?

Thank you for your comment. We agree that the original description lacked clarity regarding the loudspeaker selection process. In this experiment, time-stretched pulses (TSPs) were presented three times from each of the nine loudspeaker positions, and all recordings were made at the participant’s position. For each speaker, the recording with the least background noise among the three was selected for the analysis of impulse response. We have revised the manuscript accordingly to clarify this procedure (lines 141–144).

Line 170: It is not clear what the “reflex within its envelope” means.

We agree that the phrase “reflex within its envelope” was unclear. What we intended to convey was that, in the impulse response, the reflected sounds and reverberations are visually represented as smaller fluctuations within the overall waveform envelope. To improve clarity, we have revised the sentence as follows: “and included reverberation and reflected components that appeared as fluctuations within the waveform envelope” (lines 147, 148).

Page 8, middle: I got very confused about the various patterns – how they were characterized or used.

We acknowledge that the explanation of the analysis patterns may have been difficult to follow due to its complexity. To clarify this process, we have added Figure 2b to provide a visual example of how the acoustic waveforms were segmented using a 4–7 ms window. This figure illustrates how one of the 36 patterns was derived by shifting the start time and varying the window width. We hope that this visual representation will aid in understanding how the waveform segments were extracted and analyzed.

Line 214: The causal structure of this sentence does not make sense to me. Unless I am missing something, the number of presentations does not affect the number of different stimuli.

Thank you for pointing this out. We understand that the original sentence may have been confusing in terms of causal structure. To clarify, the number of different stimulus types was determined by the combination of three sound pressure levels (50, 55, and 60 dB SPL) and nine loudspeaker positions, resulting in 27 unique combinations. Each of these 27 stimuli was presented twice (i.e., two repetitions), yielding a total of 54 stimulus presentations. We have revised the sentence in the manuscript to improve clarity and avoid the implication of a causal relationship between the number of presentations and the number of stimulus types.

A total of 27 unique stimuli were created by combining three stimulus levels (50, 55, and 60 dB SPL) with nine loudspeaker positions. Each condition was presented twice in succession as a single trial, and participants responded once per trial. In total, 27 trials were conducted in random order (lines 198–201).

Line 251: Is this right -- the maximum number correct for each speaker and center is 21? I understood that there were 2 repetitions at each of 3 levels for each of 7 listeners (2*3*7 = 42).

We apologize for the confusion. In our experimental design, each stimulus condition—defined by a specific combination of loudspeaker position and sound pressure level (50, 55, or 60 dB SPL)—was presented twice in quick succession as a single trial. Participants responded once per trial, not per individual presentation. Therefore, for each loudspeaker, there were three sound pressure levels × 7 participants = 21 trials, resulting in a maximum of 21 correct responses per loudspeaker. We have clarified this point in the revised manuscript to ensure accuracy and to avoid misunderstandings regarding the number of responses per condition (lines 241–244).

Line 253-254: This sentence is difficult to parse. I think you mean that performance was best at 0° and decreased as the stimulus deviated from midline.

You are correct in your interpretation — we intended to convey that performance was highest at 0° and decreased as the stimulus location deviated toward the sides (±90°). We have revised the sentence accordingly to improve clarity: “The number of correct responses was highest at the 0-degree loudspeaker and gradually decreased as the stimulus location moved toward ±90 degrees” (lines 244–246).

Line 287: Consider putting information about the normal control group in a separate paragraph, for clarity.

To address the issue, we revised the Results section to clearly distinguish the analyses conducted in each group by adding the following subheadings: Control study (measurement of reflected sound), Control study (sound-localization test), and UHL study (sound-localization test) (lines 240, 282, and 299).

Line 318-319: It wasn’t clear to me how this numbering scheme affected analysis of results, which were evaluated in degrees (I think).

As our study focused on individuals with unilateral hearing loss (UHL), it was essential to account for the side of hearing loss during analysis. To avoid bias resulting from unequal numbers of participants with left- versus right-sided hearing loss, we normalized the data by aligning the deaf side to one side across participants (i.e., flipping the spatial data when necessary). This allowed us to evaluate sound-localization performance relative to the impaired side, ensuring that group-level results were not confounded by asymmetrical group sizes.

Line 359: The number of discrepancies is only informative in the context of the total number of trials. Consider reporting the percent.

Thank you for your comment. To clarify, the "more than 10 discrepancies" refers to the number of mismatched correct responses out of 21 total responses per channel. We have revised the sentence to improve clarity as follows:

“Some facilities demonstrated more than 10 discrepancies (out of 21 responses) in the number of correct responses for loudspeakers in symmetrical positions, primarily distributed between channels 1 (-90°) and 9 (90°) and channels 2 (-67.5°) and 8 (67.5°)” (lines 403–406).

Although percentage representation was considered, we opted to retain the raw values because of the small sample size per channel, where percentages could appear misleading or artificially precise.

Line 369-370: I don’t understand how this topic sentence relates to the remainder of the paragraph.

We agree that the original topic sentence did not clearly reflect the main focus of the paragraph, which centers on the relationship between early reflections and sound-localization performance. To improve coherence, we have revised the topic sentence to more accurately introduce the paragraph’s content, emphasizing the association between early reflection characteristics (e.g., envelope area and peak value) and localization accuracy (lines 381–383).

Line 383: It does not seem accurate to say that early reflections compromise the precedence effect. It might be more accurate to say that the precedence effect is stronger for longer delays.

We agree that the original phrasing may have been misleading. As you pointed out, the precedence effect is not necessarily compromised by early reflections; rather, its effectiveness depends on the temporal delay between the direct and reflected sounds. Specifically, the precedence effect is stronger when the delay exceeds a certain threshold (e.g., >1 ms for clicks), allowing the auditory system to perceptually suppress the reflection. In our study, smaller testing environments likely resulted in shorter reflection delays, potentially reducing the strength of the precedence effect and leading to greater interference in sound localization. We have revised the sentence accordingly to better reflect this interpretation (lines 393­–398).

Line 397: I may have missed it, but I don’t think this was discussed in the results.

As you noted, the association between high-frequency hearing in the better-hearing ear and azimuthal gain was discussed in the Discussion section (Line 397). In the Results section, we reported the average hearing level at 8 kHz for both ears in the UHL group, including that of the better-hearing ear (38.4 dB HL; range: 0–100 dB) (lines 306, 307). This data provides the basis for the interpretation in the Discussion that azimuthal gains were reduced when high-frequency thresholds in the better-hearing ear exceeded approximately 30 dB HL.

Line 422: This is likely to be true, but I don’t think it is accurate to say that this study showed effects of using sound-absorbing materials.

We agree with your point and acknowledge that the original wording may have overstated the conclusions of this study. Our results suggested that early reflections may negatively affect sound-localization performance. To reflect this more accurately, we have revised the sentence to state that reducing such reflections—for example, through the use of sound-absorbing materials—may help minimize inter-facility variability, rather than claiming that our study demonstrated the effectiveness of such materials (lines 435–438).

Figure 1: This figure is not needed and potentially confusing, since there isn’t really an intervention in the conventional sense.

We agree with your suggestion that Figure 1 may not be essential and could lead to confusion, as there was no intervention in the conventional sense. Accordingly, we have removed Figure 1 from the manuscript and renumbered the subsequent figures to reflect this change.
