# Supplementary material for: Establishing standardized conditions for clinically available sound-localization tests: A multicenter approach
Source: PLoS One. 2025 Aug 14;20(8):e0327124. doi: 10.1371/journal.pone.0327124 (PMC12352838; doi:10.1371/journal.pone.0327124)
Supplement: Attachment — Submitted filename: Response to Reviewers.docx [file pone.0327124.s010.docx]

6. Review Comments to the Author

Reviewer #1: Dear authors,

Thank you for having carefully adressed my comments. I have revised your new submission. I think the manuscript clarity has been improved and the methodology is clearer.

I still would like you to adress two points, linked to my previous 1) and 4) comments.

1) The correction of data availability does not appear in the new submitted manuscript (“the author(s) received no specific funding for this work “ is still mentioned).

Thank you for your comment.

Regarding the Data Availability statement, we confirm that the following sentence has already been included in the revised manuscript:

“All relevant data are within the manuscript and its Supporting Information files.”

However, we acknowledge that the Funding statement was not appropriately updated in the previous version. We have now corrected it in the manuscript to accurately reflect the funding status. The updated sentence reads:

“This study was supported by the Japan Agency for Medical Research and Development (AMED) under Grant Number JP24dk0310128.”

As it appeared that this information was also expected to be included in the cover letter, we have ensured that the updated funding statement is now clearly stated in the resubmitted cover letter.

We appreciate your careful review and apologize for the oversight.

4) lines 97-102 : Thank you for the references on poorer localization performance in individuals with UHL, and for the correction of “cognition” into “recognition”. I still do not think there is enough clarity regarding the recognition performance. It would be clearer if you mention the task you are referring to. For instance, if you are referring to speech recognition in noise, please mention a reference for that.

Thank you for your helpful comment. We understand that the term "recognition" in the original sentence may have caused confusion with speech recognition tasks. To clarify our intended meaning, we have revised the sentence to use the term “directional perception (i.e., the ability to recognize the direction of a sound source)” instead. This change more accurately reflects the context of sound localization performance in individuals with unilateral hearing loss (UHL). The revised sentence now reads:

“In general, sound-localization test results are worse in patients with unilateral hearing loss (UHL) than in healthy participants, and directional perception (i.e., the ability to recognize the direction of a sound source) on the side of the hearing-impaired ear is poorer than that on the side of the normal-hearing ear.”

We believe this clarification resolves the ambiguity and clearly indicates that the statement pertains to localization performance rather than speech recognition ability.
